# Genetic Diversity and Environmental Influence on Growth and Yield Parameters of Bambara Groundnut

**DOI:** 10.3389/fpls.2021.796352

**Published:** 2021-12-20

**Authors:** Oluwaseyi Samuel Olanrewaju, Olaniyi Oyatomi, Olubukola Oluranti Babalola, Michael Abberton

**Affiliations:** ^1^Food Security and Safety Niche Area, Faculty of Natural and Agricultural Sciences, North-West University, Mafikeng, South Africa; ^2^Genetic Resources Center, International Institute of Tropical Agriculture (IITA), Ibadan, Nigeria

**Keywords:** Bambara groundnut, genetic diversity, genotype by environment interaction, heritability, multivariate analysis, underutilized legumes, food security

## Abstract

Bambara groundnut (BGN) is a drought-tolerant crop majorly cultivated in sub-Saharan Africa. Due to a lack of extensive research, marginalization, lack of awareness, and lack of available fund among others, this crop's improvement has been limited. The development of this crop depends on evaluation and selection of unique and stable breeding lines in different environments. This study aims to estimate genetic diversity using morphological traits at different locations in 95 accessions of BGN collected from the Genebank of the International Institute of Tropical Agriculture (IITA), Ibadan. The experiment was carried out in three replicates at IITA experimental sites in two agroecological zones in Ibadan (7°40′19.62″ N, 3°91′73.13″ E) and Ikenne (6°51′00.873″ N, 3°41′48.528″ E) using a randomized complete block design. Ten vegetative growth traits and eight yield traits were scored. The data was subjected to ANOVA, PCA, correlation, and cluster analysis. Estimations of genetic parameters and broad sense heritability were carried out on the traits. ANOVA revealed significant variation in each trait except for days to emergence. Significant variation was also observed for accession and location interactions (genotype x environment interactions) for plant height, leaf length, leaf width, chlorophyll content, number of petioles, germination count, number of pods, number of seeds, seed length, seed width, and yield. PC1 and PC2 show 42.3% of the total variations observed by the PC, with seed thickness contributing more to PC1 and the number of seeds contributing more to PC2. Cluster analysis categorized the accessions into four distinct groups. The number of pods had the highest genotypic coefficient of variation of 32.55% and the phenotypic coefficient of variation of 97.61%, while seed length (0.63), seed width (0.54), and seed thickness (0.58) have high heritability values. The genetic advance was highest in yield (76.15%) and lowest in days to 50% germination (0.21%). This study can be used to predict appropriate agroecological zones for the planting of BGN while the knowledge of the diversity of the accessions based on the traits could serve a guide in selecting the best trait for the improvement of the crop.

## Introduction

Bambara groundnut (BGN) [*Vigna subterranea* (L) Verdc.] is a leguminous species that produces edible seeds. Human populations in the tropics include BGN seeds in their daily diet to compensate for the lack of proteins in their foods. Compared to other legumes, there is limited information about the extent of variation in BGN accessions (Zenabou et al., 2016). BGN represents the 3rd most important grain legume in tropical Africa. It is characterized by resistance to drought, high temperatures, and high nutrient composition. It is an indigenous African crop that has been cultivated since the 17th century (Laurette et al., 2015). It has many local names based on the area of cultivation such as jugo beans (South Africa), epa roro (Yoruba tribe in Nigeria), ntoyo ciBemba (Zambia), etc. (Olufemi, 2019). The crop is highly nutritious (Halimi et al., 2019) and grown in different countries in and out of Africa. Despite a lot of promise held from the reports of various studies (Obidiebube et al., 2020; Khan et al., 2021; Olanrewaju et al., 2021), BGN is still an underutilized crop. As a drought-tolerant legume (Mayes et al., 2019), it deserves greater attention than it is presently receiving. With the challenges of climate change affecting food production and a limited number of crops being cultivated, research into BGN should be intensified to improve the crop. Improvement of this crop will greatly impact and help in addressing the problem of food insecurity in Africa and beyond.

Most of the named cultivars have emerged majorly from the seed collection locations (Cook, 2017). It has both prostrate and erect forms and grows to a height of 0.30–0.35 m. Like the common groundnut, it has compound leaves of three leaflets which are either bunched type or spreading. The former type is self-pollinating while the latter are cross-pollinating. The branched stems of the plant root at the nodes to form a bunched herbaceous annual with a thick taproot which forms a profusion of lateral roots toward its tip (Mubaiwa et al., 2018; Valombola et al., 2019). It is an autogamous plant having pale-yellow flowers on the branching stems (Molosiwa et al., 2015; Aliyu et al., 2016). After fertilization, the stem grows into the soil with the already developed seed. The seed pod which is about 1.25–2.5 cm in diameter ends up about 1 cm beneath the soil surface having one or two seeds formed. The seeds are mostly formed after 40 days of fertilization.

The yield of any crop is a major cause for concern, especially in this era when climate change and population increase are creating challenges to sustainable food production. In finding solution to this problem, many studies have been carried out with the aim of yield improvement (Bailey-Serres et al., 2019; Oldfield et al., 2019; Schauberger et al., 2019). BGN is one of the many underutilized crops that tend to fully complement and stand at par with the major crop counterparts in terms nutrition contents (Atoyebi et al., 2017b). Hence, its importance in achieving food security. However, to fully harness its potential, good understanding of yield and yield components is needed in building an efficient breeding program for the crop.

Multi-environment trials (MET) are key for selection and recommendation (Kumar et al., 2019). MET aid in selection in terms of varieties with best yield observed in the environments under study. Hence, recommendations can be made for the most suitable environment. BGN yield is affected by soil properties and environmental factors such as rainfall and temperature. Climate change impact has not helped the production of crops globally, including BGN, thereby hampering food security (Mayes et al., 2019). Genotype x environment interactions (GEI) lead to different responses from various plant accessions for each trait. This plays a vital role in selection processes. Most farmers also grew landraces that segregated, resulting in seasonal yield variability, and lacked farmer-preferred traits like high palatability and quick processing (Mabhaudhi et al., 2013). Thus, new and improved cultivars are required to meet market demands and environmental constraints. So, improving Bambara productivity is required to justify its inclusion in diverse cropping systems. Understanding Bambara's morphological and physiological traits is the first step to efficiently exploiting its genetic diversity. This method will also help breeders and farmers by providing vital baseline data on known lines. It will also help characterize useful germplasm for breeding programs and improve selection efficiency for environmental conditions. Therefore, METs are essential in selecting high yielding accessions of crop varieties suitable for specific environments although, the adaptation of specific genotypes to various environments can be gene-specific (Olanrewaju et al., 2021). In addition, most agromorphological studies on BGN have focused on only one location with the only exception being the study by Mogale (2018).

With a view of improving BGN production, this study looks at the agromorphological variations in the various accessions reported as affected by two geographical zones in southwest Nigeria. Since these accessions originated from various sources, this study also looks at the responses in the vegetative and yield traits of these accessions based on their origin using the principal component analysis. The outcome of this study could be used to make effective strategies for the improvement of BGN for food and nutrition security.

## Materials and Methods

### Study Site Description

The study was conducted in two different agroecological zones: Ibadan (7°40′19.62″ N, 3°91′73.13″ E), which is a derived savannah, and Ikenne (6°51′00.873″ N, 3°41′48.528″ E), which is a rain forest. International Institute of Tropical Agriculture (IITA) field stations in Ibadan and Ikenne were used. The study was carried out in the 2018 and 2019 planting seasons. The average climate data for the study sites are shown in Table 1.

**Table 1:** Monthly mean meteorological data of the experimental sites during Bambara groundnut (BGN) growing season (average of 2018–2019 and 2019–2020 crop season).

			**August**	**September**	**October**	**November**	**December**	**January**
Ibadan	2018/2019	Average temperature (°C)	25	25	25	30	30	29
		Average precipitation (mm)	94.8	99.2	53.5	3.3	0	37.6
		Average relative humidity (%)	85	87	86	69	53	62
	2019/2020	Average temperature (°C)	26	26	26	28	29	29
		Average precipitation (mm)	266.7	319.8	661	69.8	1.3	0.9
		Average relative humidity (%)	82	83	85	77	62	46
Ikenne	2018/2019	Average temperature (°C)	26	26	27	28	29	29
		Average precipitation (mm)	113.2	163.9	69.3	13.3	0.6	45.1
		Average relative humidity (%)	87	90	87	86	79	82
	2019/2020	Average temperature (°C)	26	26	26	28	28	28
		Average precipitation (mm)	390.3	300.9	565.8	145.4	12.7	1
		Average relative humidity (%)	85	88	89	85	83	71

### Soil Sampling and Analysis

Topsoil samples were collected randomly from 0 to 15 cm across the plot area using the soil auger and these were bulked together to obtain a composite sample for analysis before establishing the experiment. The soil sample was dried under shade, passed through 2 mm sieve for subsequent chemical analyses [sand, clay, silt, pH, organic carbon (OC), total N, exchangeable Ca, Mg, K, available P, Na, Mn, Cu, Fe, and Zn] and particle size distribution at the onset of the experiment.

### Plant Materials

**Figure 1:**
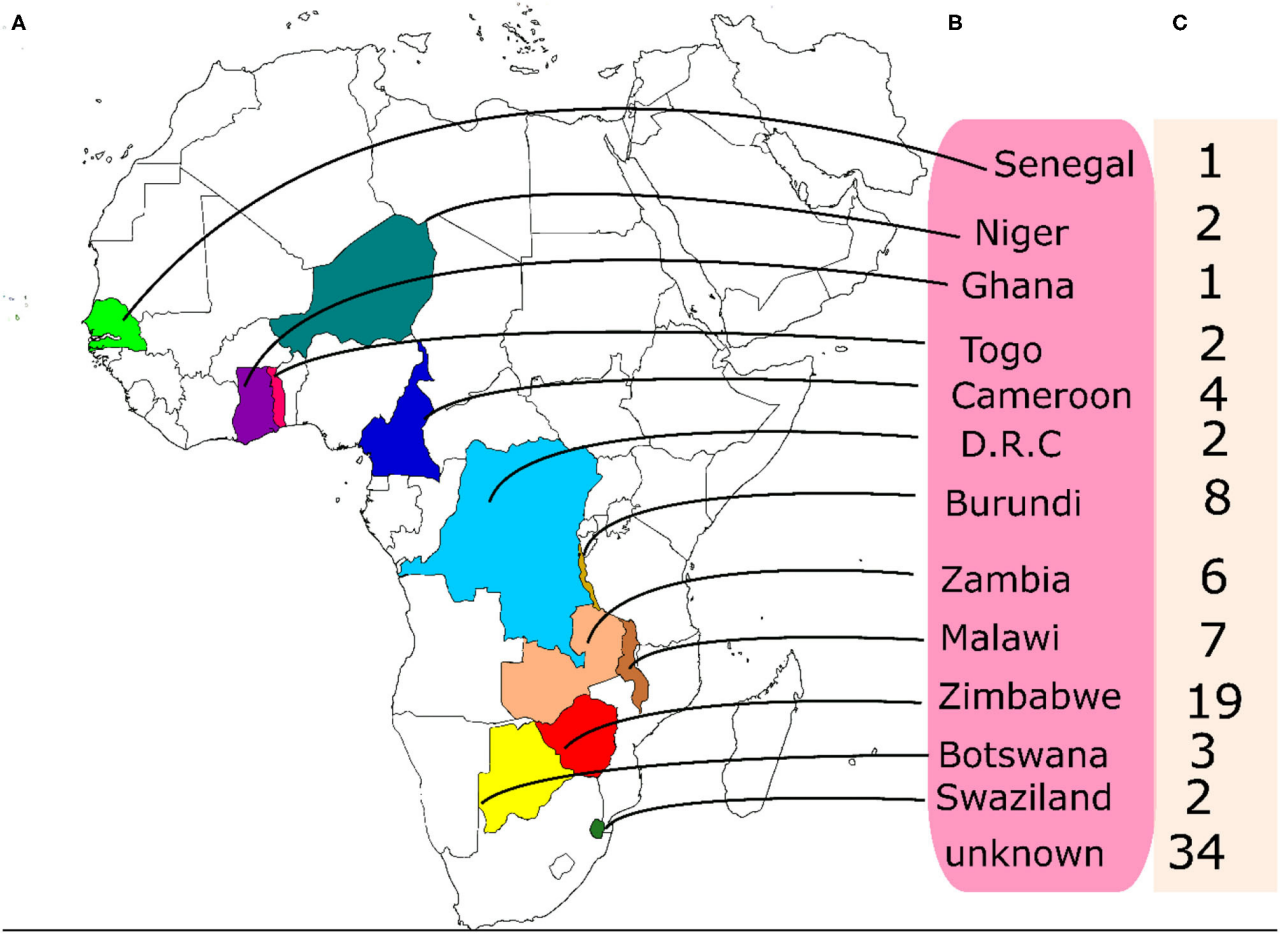
Origin and numbers of accessions from each origin used in this study. **(A)** the locations of the origin of the accessions on the map, **(B)** the names of the countries of origin **(C)** the number of accessions from each country.

A set of 95 accessions of BGN that have not been previously characterized were collected from the BGN germplasm being conserved at the Genetic Resources Center, IITA, Ibadan. The accessions originated from western to southern Africa. Out of the total number of accessions used, the origins of 34 of them were not available hence they are reported as unknown (Figure 1).

### Field Trials and Phenotyping

Randomized complete block design was used in the field trials. Sixty seeds of each accession were planted during the 2018 and 2019 planting seasons. The accessions were planted in three replicates with each replicate having 20 plants per accession on a plot which were later thinned to 10 plants at 2 weeks after emergence. The length of each plot was 3 m with 0.3 m spacing between each plant and a row spacing of 0.7 m between each plot. Each replicate contains 3 blocks which were separated by 1 m spacing and the replicates were separated from one another by 2 m spacing. The first planting in 2018 was on the 1st and 12th of September while that of 2019 season was on the 26th of August and 16th of September in Ikenne and Ibadan, respectively. Plants were rainfed until the stop of rain before irrigation was applied once in a week. According to the BGN descriptor (Ipgri/Iita/Bamnet, 2000), 10 quantitative vegetative traits were scored (Table 2).

**Table 2:** Traits scored and their abbreviations.

**Traits scored**	**Abbreviations**	**Description and time scored**
Days to emergence	DTE	Number of days from sowing to seedling emergence
Germination Count	GCT	Number of successfully established plants taken at 2 weeks after sowing
Days to 50% germination	DT50G	Number of days from sowing to when half of the seeds germinate
chlorophyll content	CHLCON	Amount of chlorophyll measured with SPAD meter before sunrise on five healthy plants at 12 weeks
Days to 1st Flower	DTF	Number of days from sowing to first flowering
Number of petioles/Accession	NPET	Recorded 12 weeks after planting; average count of petioles of five healthy plants.
Petiole length/Accession	PEL (cm)	Recorded 12 weeks after planting; average length of three leaves at the fourth node of five healthy plants
Plant height/Accession	PH (cm)	Measured from the ground level (at the base of the plant) to the tip of the highest point, including the terminal leaflet. Recorded 12 weeks after planting; average height of five plants
Leaf length/Accession	LLE (cm)	Recorded 12 weeks after planting; average length of three leaves at the fourth node of five healthy plants
Leaf width/Accession	LWI (cm)	Recorded 12 weeks after planting; average width of three leaves at the fourth node of five healthy plants
Number of pods/Accession	NPOD	Average number of 10 plants
Number of seeds/Accession	NSEED	Average number of 10 pods recorded within 2 months after harvest
Hundred seed weight	HSWT (g)	Recorded within 2 months after harvest (at 12% moisture content)
Total seeds weight/Accession	TSWT (g)	Recorded within 2 months after harvest (at 12% moisture content)
Seed length	SEEDL (cm)	Recorded within 2 months after harvest; average length of 10 seeds
Seed width	SEEDW (cm)	Recorded within 2 months after harvest; average width of 10 seeds
Seed thickness	SEEDT (cm)	Recorded within 2 months after harvest; average thickness of 10 seeds
Yield/Accession	(kg/ha)	Weight of dried seed (at 12% moisture content)

The number of pods, hundred seed weight (g), and total seed weight (g) were determined immediately after drying of the pods. The characteristics of the seeds were determined according to the method described by Saka et al. (2005). For each seed, individual seed length (L), width (W), and thickness (T) were measured using an electronic vernier caliper. T was defined as the distance from the seed's eye to the opposite end, while L and W taking in the two opposite perpendicular directions of eye seed represented the major and the minor seed diameters. Seeds were weighed to obtain a yield of the grain, which was then converted to hectare using the formula:


$$\begin{array}{l} \text{Yield}\left(\frac{\text{kg}}{\text{ha}}\right)=\frac{\text{plot yiel}{\text{d}}^{*}10000}{\text{plot area}} \end{array}$$

For each accession, a triplicate of one hundred seed weight was obtained using an electronic balance.

### Statistical Analysis

Data were checked for normality using the Shapiro-Wilk test before further analysis and all analyses were done using the R statistical package (R Core Team, 2019). The pastecs package was used to compare the responses of each trait at the various years and locations. The different traits were submitted to an ANOVA by fitting the model with the lmer function of the lme4 package in R, with all sources of variations set as random effects. Locations, blocks, replicates, accessions, and year of planting were taken as factors. The interaction effect between the genotype, year, and locations was accounted for with all sources of variations considered as random effects. Fischer's least significant difference (F-LSD) at a probability level of 5% was used to separate the means that were significantly different. PCA was done using the FactoMineR package (Lê et al., 2008) and Pearson correlation was performed using the corr. function of the stats package in R. A hierarchical cluster analysis was performed using the ward D2 method in a cluster R package (Maechler et al., 2019).

The variability was estimated using the variability package as per procedure for analysis of variance suggested by Panse and Sukhatme (1967), phenotypic coefficient variation (PCV) and genotypic coefficient variation (GCV) were calculated by the formula by Burton (1952) heritability in broad sense (h^2^) by Burton and Devane (1952) and genetic advance i.e., the expected genetic gain were calculated by using the procedure given by Johnson et al. (1955). The estimated values of PCV and GCV were categorized as described by Khan et al. (2020), 0–10% for low, 10–20% for intermediate, and ≥ 20% for high. Heritability was classified as 0–30% for low, 30–60% for intermediate, and >60% as high (Johnson et al., 1955). Genetic advance as percentage of the mean was classified as follows: 0–10% as Low, 10–20% as Moderate, and 20% and above as High. The value of k was taken as 2.06 assuming 5% selection intensity following the method of Adewale et al. (2010).

## Results

### Soil Analysis

Soil physicochemical properties for the two locations at both seasons are represented in Table 3. High amount of sand, calcium, magnesium, potassium, sodium, manganese, iron, cupper, and zinc were recorded in Ikenne compared to Ibadan for the 2018 planting season while in 2019 planting season, Ibadan had the highest pH, sand, nitrogen, organic carbon, manganese, iron, and zinc when compared with Ikenne (Table 3). Crop yield has been shown to be influenced by soil and climatic conditions. Crops respond differently to various soil types (Tolk et al., 1999). BGN produces well in sandy soils. Even though sandy soil inhibits crop emergence, BGN benefits from it because they bear fruit underground. Sandy soil has a porous structure with large pores, allowing pods to grow. When sandy soils dry out, they produce thin, loose fissures (Tester, 1990). This is an advantageous trait, especially in the semi-arid tropics where rainfall is unpredictable and soil is subjected to prolonged periods of dryness. Although clay soil has a high-water retention capacity, it expands when wet and contracts when dry over long periods of time (Brady and Weil, 2010).

**Table 3:** Soil properties at the beginning of the experiment for individual locations and seasons.

**Properties**	**2018**		**2019**	
	**Ibadan**	**Ikenne**	**Ibadan**	**Ikenne**
Sand%	73.67	80.33	79.33	75.00
Clay%	19.67	13.67	14.00	15.67
Silt%	6.67	6.00	6.67	9.33
pH	6.70	6.42	6.59	5.02
%*N*	0.17	0.10	0.10	0.09
Bray P	13.45	22.48	11.27	18.89
%OC	1.02	0.41	0.44	0.41
Ca (cmol/kg)	1.13	3.53	1.19	3.53
Mg (cmol/kg)	0.07	0.80	0.27	0.80
K (cmol/kg)	0.14	0.56	0.22	0.56
Na (cmol/kg)	0.06	0.08	0.05	0.08
Mn (ppm)	150.39	154.82	135.30	112.15
Fe (ppm)	86.22	85.84	89.46	85.58
Cu (ppm)	0.55	1.17	0.20	1.17
Zn (ppm)	1.05	1.96	2.72	1.96

### Traits Analysis

The number of petioles (20–280), chlorophyll content (14.2–99.2), hundred seed weight (0–380), and total seed weight (0–771.5) were among the traits with the largest range of values (Table 4). There was high variation among accessions for yield and yield characters, with the highest coefficient of variation (CV) observed in number of pods (93%), number of seeds (86%), and total seed weight (66%), followed by yield (39%) and hundred seed weight (36%), both showing CV <50%. These were followed by chlorophyll content (29%), leaf width (26%), days to emergence (25%), plant height (24%), petiole length (23%), and days to 50% germination (21%). The remaining traits showed CV <20%.

**Table 4:** Summary statistics of the traits scored.

	**Min**	**Max**	**Range**	**Mean**	**StdError**	**StdDev**	**CV**
DTE	3	13	10	7.63	0.06	1.88	0.25
DT50G	6	18	12	11.68	0.07	2.43	0.21
GCT	2	10	8	8.2	0.04	1.46	0.18
CHLCON	14.2	99.2	85	39.78	0.34	11.47	0.29
DTF	28	49	21	38.11	0.14	4.62	0.12
PH	7.3	48.1	40.8	25.37	0.18	6.15	0.24
LLE	3.3	12.5	9.2	6.45	0.04	1.22	0.19
LWI	0.6	7.4	6.8	2.78	0.02	0.72	0.26
PEL	2.5	33.8	31.3	16.29	0.11	3.79	0.23
NPET	20	280	260	95.74	1.25	42.13	0.44
NPOD	0	1,133	1,133	192.68	5.33	179.93	0.93
NSEED	0	1,062	1,062	184.24	4.69	158.34	0.86
HSWT	0	380	380	75.47	0.8	27.12	0.36
TSWT	0	771.5	771.5	153.51	3.01	101.7	0.66
SEEDL	2.4	17.81	15.41	11.55	0.05	1.53	0.13
SEEDW	0.34	14.58	14.24	9.17	0.03	1.14	0.12
SEEDT	0.8	14.78	13.98	9.5	0.04	1.26	0.13
YIELD	0	1266.67	1266.67	317.12	3.7	124.97	0.39

Each trait with its mean, maximum, range, standard error, standard deviation, coefficient of variation, and minimum values shown. Min, Minimum; Max, Maximum; StdError, Standard Error; StdDev, Standard Deviation; CV, coefficient of Variation. *n = 3.

Analysis of variance (ANOVA) showed that location was highly significant for all the traits (*p* < 0.0001) except for plant height (*p* < 0.001), leaf length (not significant), total seed weight (*p* < 0.05), and yield (*p* < 0.01) (Table 5). A significant block effect was observed for all traits except hundred seed weight. The accessions varied significantly in plant height, leaf length and width, days to flowering, chlorophyll content, number of petioles, germination count, petiole length, number of pods per plant, number of seeds, hundred seed weight, total seed weight, seed length, seed width, seed thickness, and yield (*p* < 0.0001) while days to 50% germination (*p* < 0.001) and days to emergence (*p* < 0.01) were also significant. Remarkably, location and accession interaction effect were highly significant on plant height, chlorophyll content, number of petioles, germination count, number of pods, and number of seeds (*p* < 0.0001), while leaf length, leaf width, and yield were significant at *p* < 0.001, days to 50% germination and seed length were significant at *p* < 0.01 and at *p* < 0.05, days to emergence, petiole length, and seed width were significant. Furthermore, interaction between accession and year was highly significant for plant height, number of pods, and number of seeds (*p* < 0.0001), leaf length (*p* < 0.001), and leaf width (*p* < 0.01). There was a high significant effect of location, accession, and year interaction on leaf length, leaf width, petiole length, number of pods and seeds (*p* < 0.0001), plant height, and days to flowering (*p* < 0.01). The mean comparison from the LSD analysis for each accession, location, and the year is shown in Supplementary Table S1. The result in Supplementary Table S1 showed that high level of variability and heterogeneity exist among accessions, locations, and years in response to the traits scored.

**Table 5:** ANOVA result for traits scored.

**SOV**	**Df**	**PH**	**LLE**	**LWI**	**DTF**	**DTE**	**CHL**	**NPET**	**GCT**	**PEL**	**DT50G**	**NPOD**	**NSEED**	**HSWT**	**TSWT**	**SL**	**SW**	**ST**	**YLD**
Lctns(L)	1	120.9^**^	1.04^ns^	7.27^***^	8702.0^***^	342.04^***^	18556.6^***^	189,875^***^	264.0^***^	498.33^***^	1709.1^***^	549,782^***^	1,215,454^***^	20,048^***^	22,326^**.**^	135.59^***^	40.76^***^	72.68^***^	77,311^*^
Block	4	1019.5^***^	26.16^***^	7.22^***^	147.0^***^	9.74^*^	212.5^.^	17,465^***^	7.26^***^	194.87^***^	16.98^**^	1,003,097^***^	56,0362^***^	812 ^ns^	224,046^***^	4.53^*^	5.94^***^	3.02^*^	57,470^**^
Rep	2	0.5	1.70	0.65	23.4	168.94	2697.9	3,033	7.50	42.02	57.36	12,005	4,413	355	8,930	0.67	0.21	0.94	27,826
Accns(A)	94	83.0^***^	2.12^***^	1.14^***^	6.4^***^	4.17^*^	239.9^***^	3515^***^	3.75^***^	26.22^***^	6.63^**^	50,848^***^	35,141^***^	1,421^***^	11,576^***^	9.96^***^	4.56^***^	6.09^***^	28,482^***^
Year(Y)	1	9222.4^***^	187.91^***^	33.10^***^	2528.8^***^	0.55^ns^	87.0^ns^	113,973^***^	180.67^***^	1172.03^***^	4.01^ns^	4,597,305^***^	3,773,330,440^***^	47,969^***^	876,694^***^	13.84^**^	40.85^***^	7.14^**^	96,718^**^
L^*^A	94	23.1^***^	1.27^**^	0.48^**^	4.5^ns^	3.74^.^	215.1^***^	2,231^***^	4.14^***^	10.21^.^	5.76^*^	23,977^***^	18,538^***^	490^ns^	6,030^ns^	1.82^*^	1.04^.^	1.10^ns^	21,245^**^
L^*^Y	1	6677.8^***^	165.09^***^	39.27^***^	7490.2^***^	0.50 ^ns^	81.0^ns^	108,052^***^	166.88^***^	1192.74^***^	0.03^ns^	8,077,471^***^	5,127,831^***^	16,767^***^	1,963,188^***^	43.27^***^	34.13^***^	67.07^***^	6532^ns^
A^*^Y	94	23.2^***^	1.30^**^	0.41^*^	4.2^ns^	1.59 ^ns^	21.07^ns^	682^ns^	0.62^ns^	9.90^ns^	1.82^ns^	28,598^***^	24,255^***^	536^ns^	7234^ns^	1.48^ns^	1.02^ns^	1.18^ns^	8,855^ns^
L^*^A^*^Y	94	18.5^**^	1.58^***^	0.52^***^	5.8^**^	1.63^ns^	21.6^ns^	695^ns^	0.52^ns^	20.96^***^	1.82^ns^	22,755^***^	19,397^***^	690 ^ns^	7230^ns^	1.28^ns^	0.8^ns^	1.05^ns^	14,147^ns^
Res	754	12.0	0.87	0.32	4.0	3.0	103.7	1,146	1.23	8.42	4.441	10,284	9,927	602	6,617	1.42	0.85	1.0	13,909
GM		25.39	0.82	0.44	1.58	0.87	1.6	1.94	0.9	1.23	1.06	6.81	6.89	6.21	6.78	3.52	3.18	3.23	8.26
LSD		2.78	0.75	0.45	1.60	1.39	8.16	27.13	0.89	2.32	1.68	81.28	79.85	19.67	65.19	0.95	0.74	0.80	94.52

*Trait values indicate the mean square values; Lctns, locations; Accns, accessions; Res, residuals; GM, grandmean; Df, degree of freedom; CHL, chlorophyll content; SL, seed length; SW, seed width; ST, seed thickness; YLD, yield; other traits are as represented in Table 2*.

*** *Highly significant at p < 0.0001*.

** *Highly significant at p < 0.001*.

* *Highly significant at p < 0.01*.

. *Significant at p < 0.05*.

*ns, no significance*.

### Principal Component Analysis

**Figure 2:**
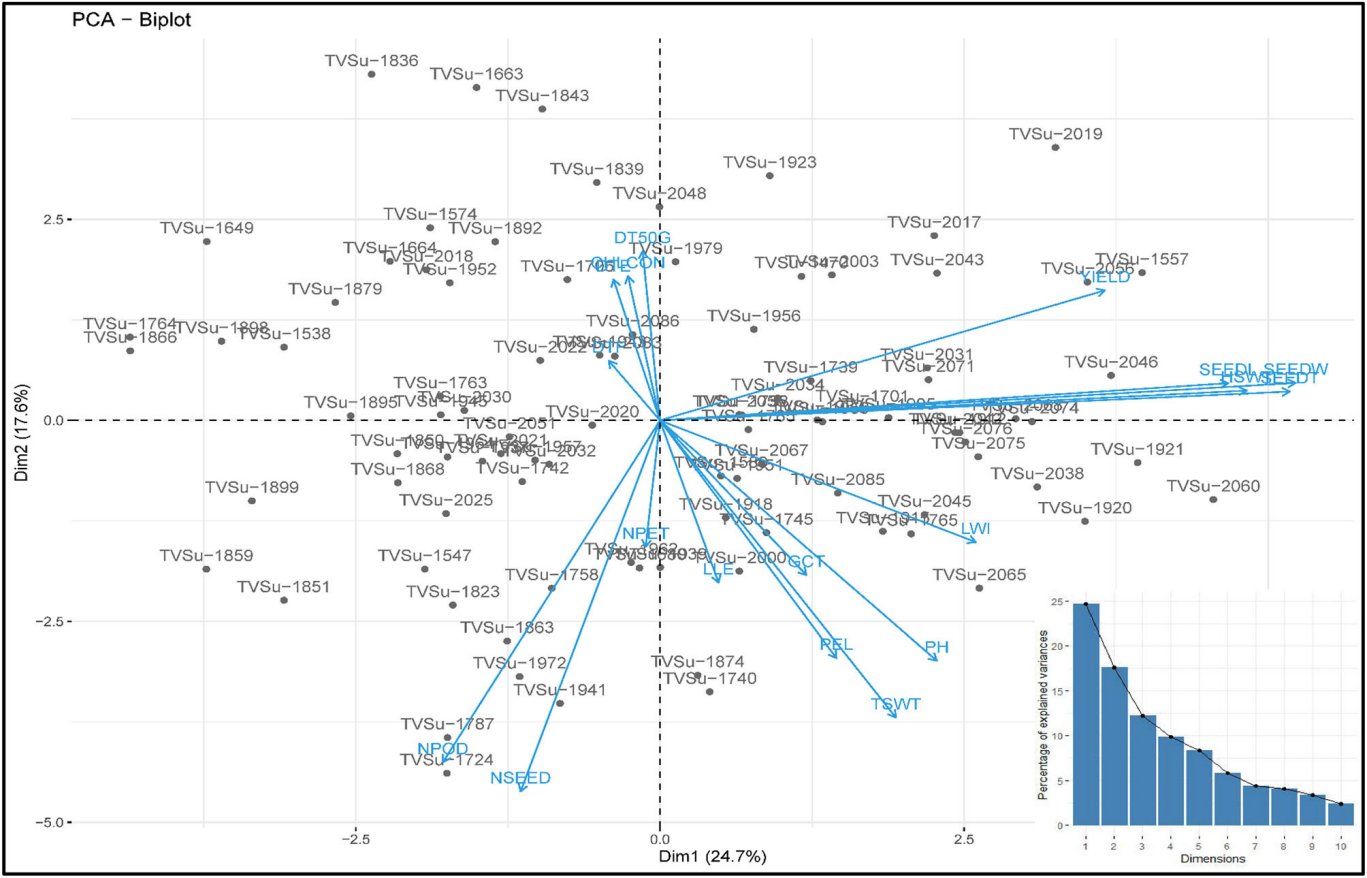
PC 1 and PC 2 biplot using quantitative trait scores of the Bambara groundnut (BGN) accessions. Each accession is represented according to its origin. Inset is the scree plot showing the explained variances.

The principal component of the variances taken by each accession and the overall component response of the accessions on the traits over the environments were represented in a biplot (Figure 2). PC1 (Dim1) and PC2 (Dim2) accounted for 42.3% of the total variances observed. PC1 accounted for 24.67% of the total variations while PC2 accounted for 17.63%. PCA biplot loading both variables and accessions showed how strongly each trait influences a PC and how they are correlated to each other. The lesser angle between two vectors (Supplementary Figure S6) indicated higher and positive correlation (e.g., NPOD and NSEED, SEEDW, and SEEDT). However, when angle between two vectors form 90°, it indicated no correlation and when it is more than 90°-180°, it indicated negative correlation between the traits (e.g., DTF and PH). Among the variables, seed width (19.53%), seed thickness (19.58%), hundred seed weight (16.98%), seed length (15.93%), and yield (9.76%) were the major contributing traits in PC1 while number of seeds (21.78%), number of pods (18.48%), total seed weight (13.96%), plant height (9.12%), and petiole length (8.93%) had the highest contributions to PC2 (Table 6; Supplementary Figures S3–S7). The contributions of each trait to all components were represented in Supplementary Figures S1–S3. The individual PCA plot (Supplementary Figure S7) showed that most of the accessions were dispersed at low distances while few were dispersed at high distances as reflected by eigenvector (Table 6). From the biplot, we can conclude that the accessions loading on PC1 were high yielding with thick and long seeds while at the same time having high hundred seed weight. Accessions loading on PC2 have high number of seeds, number of pods, and total seed weight. Furthermore, these accessions on PC2 were tall plants with longer petioles (Figure 2).

**Table 6:** Trait contributions, eigenvalues, and cumulative percentage of the components.

	**PC1**	**PC2**	**PC3**
PH	3.78	9.12	10.80
LLE	0.17	4.16	10.57
LWI	4.92	2.34	11.87
DTE	0.11	3.14	5.74
DT50G	0.01	4.53	3.55
CHLCON	0.05	3.29	0.09
GCT	1.06	3.78	1.33
DTF	0.13	0.56	21.25
NPET	0.01	2.53	3.87
PEL	1.54	8.93	14.92
NPOD	2.33	18.48	2.93
NSEED	0.96	21.78	1.00
HSWT	16.98	0.14	3.98
TSWT	2.75	13.96	4.96
SEEDL	15.93	0.22	0.16
SEEDW	19.93	0.22	0.32
SEEDT	19.58	0.13	0.34
YIELD	9.76	2.68	2.30
Eigenvalue	4.44	3.17	2.19
Percentage variance	24.67	17.63	12.19
Percentage cumulative variance	24.67	42.3	54.49

*PH, plant height (cm); LLE, leaf length (cm); LWI, leaf width (cm); DTE, days to emergence; DT50G, days to 50% germination; CHLCON, chlorophyll content; GCT, germination count; DTF, days to flowering; NPET, number of petioles; PEL, petiole length (cm); NPOD, number of pods; NSEED, number of seeds; HSWT, hundred seed weight (g); TSWT, total seed weight (g); SEEDL, seed length (cm); SEEDW, seed width (cm); SEEDT, seed thickness (cm), Yield (kg/ha)*.

### Cluster Analysis

**Figure 3:**
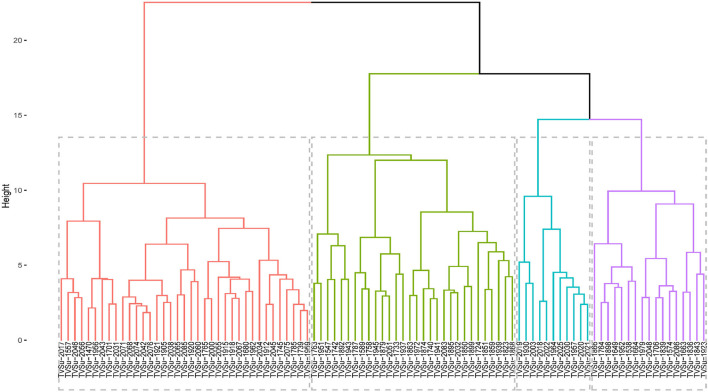
Hierarchical clustering dendrogram analysis; Euclidean distance was used and the associations between groups were done by the Ward method for morphological traits across the environments.

The accessions were grouped into 4 groups based on the agromorphological traits (Figure 3), with the clusters in red having the highest number of accessions (37 accessions) followed by the ones in green (30), blue had 11, and the purple cluster with 17 accessions. The clusters in red consisted of TVSu-2017, TVSu-1557, TVSu-2046, TVSu-2056, TVSu-1470, TVSu-1956, TVSu-2043, TVSu-1701, TVSu-2031, TVSu-2071, TVSu-2068, TVSu-2074, TVSu-2042, TVSu-2076, TVSu-1921, TVSu-1905, TVSu-2038, TVSu-2065, TVSu-2085, TVSu-1920, TVSu-2060, TVSu-1765, TVSu-2000, TVSu-2055, TVSu-1915, TVSu-1918, TVSu-2067, TVSu-1680, TVSu-1962, TVSu-2034, TVSu-1912, TVSu-2045, TVSu-1745, TVSu-2075, TVSu-1785, TVSu-1739, and TVSu-1959 while the green cluster is made up of TVSu-1763, TVSu-1951, TVSu-1547, TVSu-1742, TVSu-1892, TVSu-1943, TVSu-1787, TVSu-1589, TVSu-1758, TVSu-1945, TVSu-1879, TVSu-2051, TVSu-1733, TVSu-1937, TVSu-1863, TVSu-1972, TVSu-1874, TVSu-1740, TVSu-1941, TVSu-2083, TVSu-1895, TVSu-2032, TVSu-1850, TVSu-1899, TVSu-1724, TVSu-1851, TVSu-1859, TVSu-1939, TVSu-1823, and TVSu-1868, and the blue cluster consisted of TVSu-2019, TVSu-1930, TVSu-2003, TVSu-2018, TVSu-2022, TVSu-1964, TVSu-2025, TVSu-2030, TVSu-1957, TVSu-2020, and TVSu-2021. Finally, the purple cluster consisted of TVSu-1866, TVSu-1764, TVSu-1898, TVSu-1649, TVSu-1952, TVSu-1538, TVSu-1664, TVSu-1979, TVSu-2048, TVSu-1706, TVSu-1839, TVSu-1574, TVSu-2086, TVSu-1663, TVSu-1836, TVSu-1843, and TVSu-1923 (Figure 3).

Accessions in the red cluster were characterized by high mean values for days to emergence, plant height, seed thickness, seed width, seed length, hundred seed weight, and yield while those in the green cluster were characterized by high mean values for number of petioles, number of pods, number of seeds, and total seed weight. On the other hand, the blue and purple clusters were both characterized by accessions with low leaf width, plant height, number of pods, number of seeds, hundred seed weight, and total seed weight. Interestingly, the red cluster is dominated with accessions whose origins are not known (Supplementary Table S4). We can say that these accessions might be from the same origin or region. Other accessions present in this cluster are from Burundi (1), Cameroon (3), DRC (1), Ghana (1), Togo (2), Malawi (2), Zambia (2), and Zimbabwe (3). Remarkably, the green cluster consist of accessions from the southern region of Africa which include Botswana, Malawi, Swaziland, Zambia, and Zimbabwe. Furthermore, accessions from Zimbabwe are more dominant in this cluster. Like the green cluster, the blue cluster is also dominated by accessions from southern Africa with Burundi having the highest number of accessions in this cluster. Finally, the purple cluster largely consists of accessions from the western part of Africa with Senegal producing the highest number of accessions (Supplementary Table S4). Hence, accessions from the red clusters which are majorly from unknown origin are more desirable for selection for improved breeding programs while those in the green cluster, dominated by accessions from southern Africa, can also be considered.

### Correlation Analysis of the Traits

**Figure 4:**
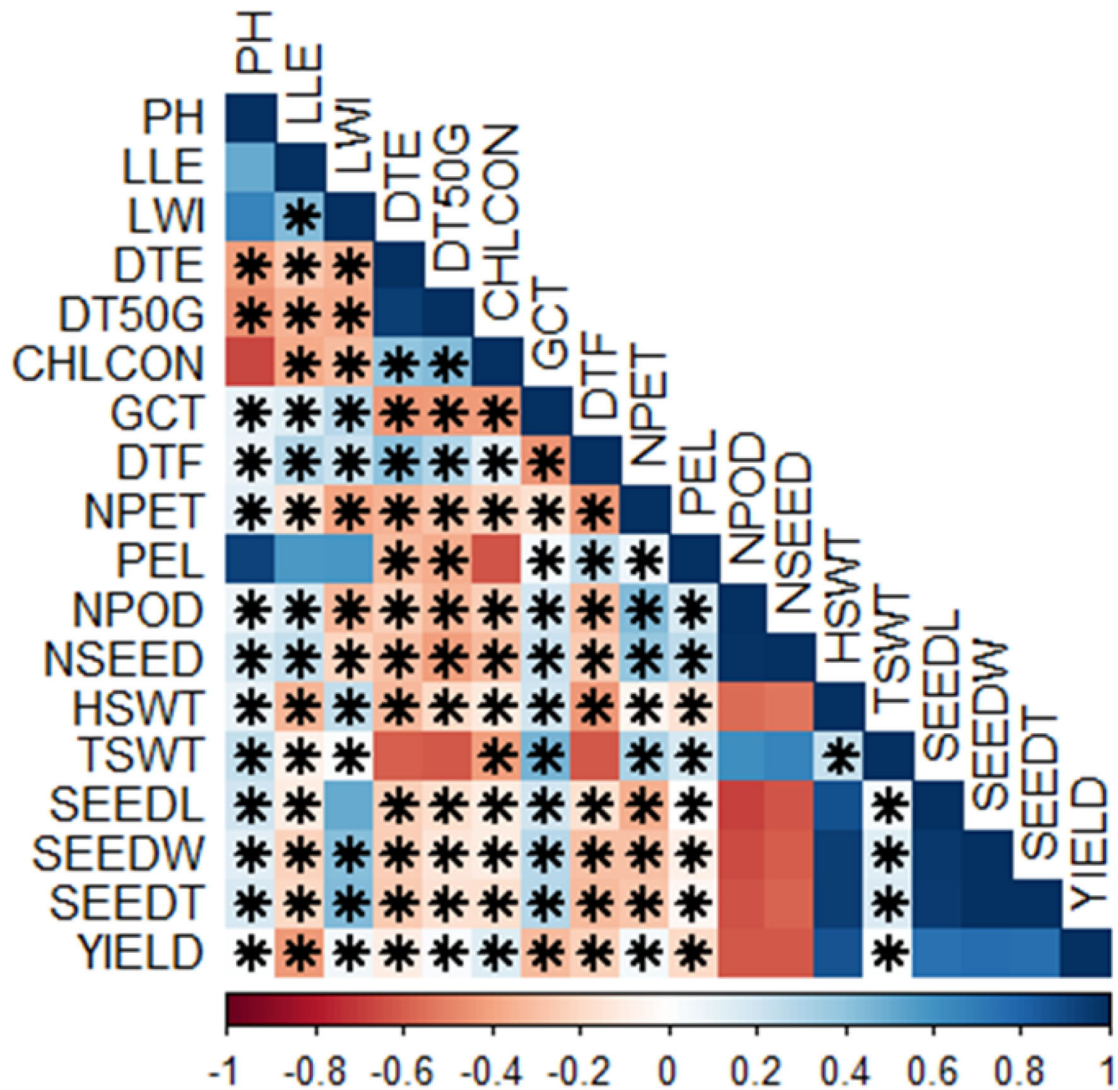
Correlations among the traits scored; Pearson's rank correlation matrix and performance analytic chart of the variables showing the relationship among the variables scored on BGN accessions grown across two locations and years. (Blue color indicates a significant positive correlation and red color indicates a significant negative correlation among different traits. PH, plant height (cm); LLE, leaf length (cm); LWI, leaf width (cm); DTE, days to emergence; DT50G, days to 50% germination; CHLCON, chlorophyll content; GCT, germination count; DTF, days to flowering; NPET, number of petioles; PEL, petiole length (cm); NPOD, number of pods; NSEED, number of seeds; HSWT, hundred seed weight (g); TSWT, total seed weight (g); SEEDL, seed length (cm); SEEDW, seed width (cm); SEEDT, seed thickness (cm), Yield (kg/ha). *Significant, *p* < 0.05.

There were lots of significant correlations among the traits scored (Figure 4). The level of significance of the correlations at *p* < 0.05 is shown by the asterisks. Either positive or negative correlations, the asterisks indicate if it is statistically significant or not. The red colors showed negative correlations while the blue colors showed positive correlations. The deeper the colors, the stronger the correlations. The correlation matrix showed that plant height had a positive correlation with leaf length (*r* = 0.51, *p* < 0.05), leaf width (*r* = 0.67, *p* < 0.01), germination count (*r* = 0.09, *p* = 0.72), days to flowering (*r* = 0.09, *p* = 0.73), number of petioles (*r* = 0.12, *p* = 0.64), petiole length (*r* = 0.92, *p* < 0.001), number of pods (r = 0.07, *p* = 0.78), number of seeds (*r* = 0.17, *p* = 0.5), hundred seed weight (*r* = 0.09, *p* = 0.72), total seed weight (*r* = 0.24, *p* = 0.34), seed length (*r* = 0.19, *p* = 0.44), seed width (*r* = 0.12, *p* = 0.63), seed thickness (*r* = 0.16, *p* = 0.52), and yield (*r* = 0.00, *p* = 0.96) but negatively correlates with the remaining traits (Supplementary Tables S2, S3). Among the positive correlations however, correlations with leaf length, leaf width, petiole length, and chlorophyll content were not significant (Supplementary Tables S2, S3). Leaf length, in addition to plant height, positively correlated with leaf width (*r* = 0.43, *p* = 0.07), germination count (*r* = 0.14, *p* = 0.59), days to flowering (*r* = 0.29, *p* = 0.24), petiole length (*r* = 0.57, *p* < 0.01), number of pods (*r* = 0.16, *p* = 0.52), and number of seeds (*r* = 0.23, *p* = 0.35) but correlation with petiole length is not significant (Supplementary Tables S2, S3). Furthermore, leaf width correlated positively with germination count (*r* = 0.28, *p* = 0.27), days to flowering (*r* = 0.22, *p* = 0.38), petiole length (*r* = 0.59, *p* < 0.01), hundred seed weight (*r* = 0.25, *p* = 0.32), total seed weight (*r* = 0.02, *p* = 0.95), seed length (*r* = 0.50, *p* < 0.05), seed width (*r* = 0.42, *p* = 0.08), seed thickness (*r* = 0.44, *p* = 0.07), and yield (*r* = 0.06, *p* = 0.82), while days to emergence also had a positive correlation with days to 50% germination (*r* = 0.94, *p* < 0.001), chlorophyll content (*r* = 0.40, *p* < 0.5), and days to flowering (*r* = 0.40, *p* < 0.5). Days to 50% germination, on the other hand, correlated positively with chlorophyll content (*r* = 0.43, *p* = 0.07), days to flowering (*r* = 0.29, *p* = 0.24), and yield (*r* = 0.02, *p* = 0.92) while chlorophyll content correlated positively with days to flowering and yield (Supplementary Tables S2, S3). In addition, germination count had a positive correlation with petiole length (*r* = 0.03, *p* = 0.89), number of pods (*r* = 0.18, *p* < 0.5), number of seeds (*r* = 0.22, *p* < 0.5), hundred seed weight (*r* = 0.19, *p* < 0.5), total seed weight (*r* = 0.46, *p* ≤ 0.05), seed length (*r* = 0.19, *p* < 0.5), seed width(*r* = 0.28, *p* < 0.5), and seed thickness (*r* = 0.29, *p* < 0.5), while days to flowering correlated positively with petiole length (*r* = 0.25, *p* < 0.5). Number of petioles had a positive correlation with petiole length (*r* = 0.06, *p* = 0.8), number of pods (*r* = 0.43, *p* = 0.07), number of seeds (*r* = 0.39, *p* = 0.11), total seed weight (*r* = 0.31, *p* = 0.21), and yield (*r* = 0.05, *p* = 0.85) while petiole length had positive correlation with number of pods (*r* = 0.17, *p* = 0.51), number of seeds (*r* = 0.23, *p* < 0.5), and total seed weight (*r* = 0.17, *p* < 0.5). Number of pods positively correlated with number of seeds (*r* = 1.0, *p* < 0.001) and total seed weight (*r* = 0.62, *p* < 0.01); number of seeds positively correlated with number of petioles (*r* = 0.39, *p* < 0.5) and total seed weight (*r* = 0.67, *p* < 0.01). Hundred seed weight had positive correlations with total seed weight (*r* = 0.23, *p* < 0.5), seed length (*r* = 0.88, *p* < 0.001), seed width (*r* = 0.95, *p* < 0.001), seed thickness (*r* = 0.94, *p* < 0.001), and yield (*r* = 0.86, *p* < 0.001); total seed weight had a positive correlation with seed length (*r* = 0.03, *p* = 0.89), seed width (*r* = 0.14, *p* = 0.59), seed thickness (*r* = 0.16, *p* = 0.53), and yield (*r* = 0.00, *p* = 0.99). Seed length had positive correlation with seed width (*r* = 0.96, *p* < 0.001), seed thickness (*r* = 0.96, *p* < 0.001), and yield (*r* = 0.74, *p* < 0.001), while seed width had a positive correlation with seed thickness (*r* = 1.0, *p* < 0.001) and yield (*r* = 0.77, *p* < 0.001). Finally, seed thickness had positive correlation with yield (*r* = 0.76, *p* < 0.001) in addition to the other correlations earlier reported (Supplementary Tables S2, S3; Figure 4).

### Analysis of Genetic Components

The output of genetic components analysis was compiled in Table 7. Expectedly, the phenotypic variance was higher than the genotypic variance in all the traits. Yield (kg ha^−1^) reported higher phenotypic (19,476.39) and genotypic (5,159.09) variances, while the lower phenotypic (0.68) and genotypic (0.23) variances were observed in leaf width. The traits, such as leaf length (LLE; GCV 7.18, PCV 19.95), germination count (GCT; GCV 6.50, PCV 19.61), days to flowering (DTF; GCV 6.31, PCV 10.81), seed length (SEEDL; GCV 14.41, PCV 18.19), seed width (SEEDW; GCV 11.84, PCV 16.13), and seed thickness (SEEDT; GCV 13.48, PCV 17.66), showed below 20% of PCV and GCV. Improvement of this crop can be based on traits with GCV ≥ 20% [number of petioles (NPET), number of pods (NPOD), hundred seed weight (HSWT), and Yield) which indicated high variability among these traits although they are influenced by additive genes. Due to the lower GCV values (≤ 10%), LLE, days to emergence (DTE), days to 50% germination (DT50G), GCT, and DTF indicated the limited chance of selection due to environmental effect on their phenotypic expression.

**Table 7:** Variance components, heritability, and genetic advance for growth and yield traits.

**Traits**	**V_**P**_**	**V_**G**_**	**GCV (%)**	**PCV (%)**	**h^**2**^ (%)**	**GA**	**GAM**
PH	48.56	14.21	14.86	27.47	29	4.20	16.56
LLE	1.66	0.21	7.18	19.95	13	0.32	5.33
LWI	0.68	0.23	16.74	29.65	32	0.54	19.48
DTE	3.48	0.31	7.33	24.47	9	0.35	4.52
DT50G	6.00	0.25	4.32	20.97	4	0.21	1.83
CHLCON	159.36	42.84	16.45	31.74	27	6.99	17.57
GCT	2.58	0.61	6.50	19.61	23	0.78	9.47
DTF	16.99	5.78	6.31	10.81	34	2.89	7.57
NPET	2200.51	569.26	24.92	49.00	26	25.00	26.11
PEL	17.45	4.12	12.46	25.64	24	2.03	12.47
NPOD	35373.05	3932.95	32.55	97.61	11	43.08	22.36
NSEED	26053.10	1256.54	19.24	87.61	5	16.04	8.70
HSWT	923.32	248.87	20.90	40.26	27	16.87	22.35
TSWT	9870.43	631.61	16.37	64.72	6	13.10	8.53
SEEDL	4.41	2.77	14.41	18.19	63	2.72	23.53
SEEDW	2.19	1.18	11.84	16.13	54	1.64	17.89
SEEDT	2.81	1.64	13.48	17.66	58	2.01	21.20
YIELD	19476.39	5159.09	22.65	44.01	26	76.15	24.01

*V_G_, genotypic variance; V_P_, phenotypic variance; h^2^, heritability in broad sense; PCV, phenotypic coefficient of variation; GCV, genotypic coefficient of variation; GA, genetic advance; GAM, genetic advance of the mean; PH, plant height (cm); LLE, leaf length (cm); LWI, leaf width (cm); DTE, days to emergence; DT50G, days to 50% germination; CHLCON, chlorophyll content; GCT, germination count; DTF, days to flowering; NPET, number of petioles; PEL, petiole length (cm); NPOD, number of pods; NSEED, number of seeds; HSWT, hundred seed weight (g); TSWT, total seed weight (g); SEEDL, seed length (cm); SEEDW, seed width (cm); SEEDT, seed thickness (cm), Yield (kg/ha)*.

The values of heritability in broad sense were low for most of the traits (Table 7), which ranged from 4% (DT50G) to 63% (SEEDL). Very high (≥ 60%) heritability was measured for SEEDL (63%), indicating that this is the only trait that was highly heritable in this study. The heritability value 30–60% was marked for leaf width (LWI, 32%), DTF (34%), SEEDW (54%), and SEEDT (58%) which indicated that the traits were moderately heritable whereas plant height (PH, 29%), HSWT (27%), yield (26%), and the remaining traits showed heritability below 30%, i.e., low heritability. The trait NPET (26.11%) had highest genetic advance (as percentage mean) value (≥ 20%) while the lowest was 1.83% for DT50G (Table 7). Remarkably, the higher genetic gain was recorded for grain yield (76.15%), and NPODS (43.08%).

## Discussion

High nutrient uptake, the ability to compete favorably with weeds, and yield improvement are some of the prerequisites for developing high yield crops. The significant differences for the different traits scored on the accessions indicate the existence of variability in the selected population that can be exploited for an improved breeding program. Trait variability facilitates trait-assisted selection of best lines for improvement (Moradi et al., 2019; Dewi et al., 2020). Various studies have shown GEI effect on several crops, such as Rice (Rahman and Shah, 2019; Calayugan et al., 2020), sweet potato (Ngailo et al., 2019), and Sorghum (Jiang et al., 2020). Yan and Kang (2003) stated that the number of genotypes and environments determines the extent of environmental variation. They also pointed out that many genotypes in few numbers of environments reduce the environmental variance and *vice versa*. However, according to Aremu et al. (2019), the environment is always the dominant source of variation, and it must be prioritized in plant breeding. Plant germplasm possesses a high degree of variability in its morphology. This high range of variability is harnessed to develop improved cultivars in major crops. Less known or underutilized crops need to benefit from this development too, as they also have a high level of diversity between (inter-variation) and within (intra-variation) their accessions. These are, however, further seen in the effects of various locations where they are cultivated. Therefore, morphological characterization in various locations assists breeders in selecting superior lines for further improvement (Peratoner et al., 2016; Moradi et al., 2019; Dewi et al., 2020).

Bambara groundnut (BGN) is a rich source of diversity as it has been localized in various environments and its relevance, especially in sub-Saharan Africa, is increasing greatly. In this study, all accessions used showed a high level of diversity for all traits studied. This result supports that of other studies that report high variability in BGN accessions (Mohammed et al., 2016; Atoyebi et al., 2017a; Gbaguidi et al., 2018). In the studies carried out by Mohammed et al. (2016), Gbaguidi et al. (2018), and Atoyebi et al. (2017a), the authors recorded CV ≥ 20% for petiole length, number of pods, hundred seed weight, and yield. The high coefficient of variation observed in some of the traits in this study shows a high level of heterogeneity for these traits among the accessions studied (Table 4). High heterogeneity in BGN was also reported by Goli et al. (1997), Khan et al. (2020), and Khan et al. (2021). Diversities in morphology result from differences in the genetic make-up (Manimekalai et al., 2018; Ibrahim et al., 2019) of the crop and the likely impact of the locations and planting seasons. We can also attribute these diversities to the inconsistencies observed in traits like DTF, which are affected by day-light length. In this study, the reported DTF were between 28 and 49 days, but Khan et al. (2021) reported 36–53 days, Masindeni (2006) reported 43–80 days, and Goli et al. (1997) reported 38–68 days.

Genotype x environment interactions (GEI) reduces the relationship between genotype and phenotype (Voss-Fels et al., 2019), thereby reducing the progress of selection. Hence, there is a need to study GEI for an effective breeding program for BGN to enable breeders to identify locations that are good representatives of target regions of interest (Gupta et al., 2015). Plant genotypes often tested in different locations and years are affected by differences in soil fertility, climatic factors (such as rainfall, humidity, and temperature), pests, and diseases. This study showed that plant growth-trait responses, especially the architectural traits, are affected by accessions, locations, and years (Table 5). This finding is supported by the result obtained in the study carried out by Mogale (2018) at three different locations in South Africa. The accessions responded differently to each trait at the different locations and years, thus suggesting the existence of GEI (Supplementary Table S1). The highly significant location effect observed for all traits (except plant height and leaf length), can be attributed to differences in the climatic and soil conditions exhibited at the two locations (Tables 2, 3). This suggests the importance of assessment of accessions under different environments to identify better performing accessions. Effects of GEI on crop evaluation have been reported in various studies (Aarthi et al., 2020; Rubilar et al., 2020; Olanrewaju et al., 2021). However, the accessions showed significant variation in plant height, which is contrary to the reports of Ntundu et al. (2006) in Tanzania and Shegro et al. (2013) in South Africa. The yield and yield-related traits in this study showed a high genetic discrepancy. A similar report was given by Shegro et al. (2013), and these variations were accredited to the effect of genotype by environment interaction on BGN yield. The hundred seed weight is critical for determining morphological traits related to plant yield (Gerrano et al., 2015; Khan et al., 2021). The yield of BGN was recorded from 146.6 to 2678.6 kg ha^−1^ by Gbaguidi et al. (2018) and 1,058.8 kg ha^−1^ by Dansi et al. (2012), whereas in this study, we report from 0 to 1,266.67 kg ha^−1^. Typically, the Food and Agriculture Organization (FAO; http://www.fao.org/faostat/en/#data/QC) estimated the average yield of BGN (1,180 kg ha^−1^) is lower than our estimated yield. Variation in seed length and seed width, as observed in this study, may be due to different seed shapes, while variation in hundred seed weight can be attributed to different seed sizes. The findings from this study and other studies show a high level of diversity and a high influence of the environment on the growth and yield of BGN.

Different algorithms, such as multidimensional scaling, clustering, and PCA, are used to assess variability and genetic diversity in various studies (Franco-Duran et al., 2019; Zarei et al., 2019; Munir et al., 2020). In PCA biplot, traits are superimposed on the plot as vectors. Biplot represents the association among different traits and accessions, and the length of the vectors show the contribution of each trait to the observed variations. The eigenvalues and the corresponding factors are sorted by descending order of how much of the initial variability they represent. The Eigenvalue significance criterion, as described by Kaiser (1960), was used to select statistically significant principal components. Among 10 components, the 2 selected showed a value of more than 1. The highest variation observed is represented in the first axis (Iezzoni and Pritts, 1991). In this study, the first component (PC1) accounted for a greater proportion of the variation than the second component (Table 6; Figure 2). Similar results were reported by Khan et al. (2021) of total variations at 45.88% (PC1) and 10.68% (PC2) in BGN. Several studies also support these findings (Farhad et al., 2008; Usman et al., 2014; Atoyebi et al., 2017a; Mohammed et al., 2020). In addition, PC1 is the most powerful criterion for selection for yield improvement (Adeoti et al., 2012). In this study, leaf width, hundred seed weight, seed width, seed thickness, seed length, and yield have more influence on PC1. Similar findings were reported by Stoilova and Pereira (2013), Ridzuan et al. (2019), and Khan et al. (2021).

From the biplot (Figure 2), accessions that are tall have longer petioles and longer and wider leaves, but these same accessions are low in chlorophyll content and number of petioles. They do not germinate well-compared to the other accessions in the other principal components, they emerge late, and take a longer time to flower and *vice versa*. This study did not determine whether these tall accessions with longer petioles and longer and wider leaves take longer to mature. But we can conclude from the findings that the tall plants should attain maturity earlier than the short plants because the tall plants flower early, which means that they enter the vegetative stage earlier than the short plants. Similar correlations have been reported in Chickpea where the tall plants flower earlier than the short plants (Mallikarjuna et al., 2019). Likewise, accessions that have a higher number of petioles and leaves, flower late, and have poor germination. Chlorophyll content correlates negatively with plant height, but chlorophyll aids in the process of photosynthesis. It might be expected that tall plants have high chlorophyll content. However, short plants with more green leaves will have more chlorophyll content than tall plants with fewer green leaves. Hence, the number of green leaves, not the height of a plant, should be used to determine the chlorophyll content of a plant. This result is supported by the findings in the studies of Anwar et al. (2020) and Klaassen et al. (2020). They reported that taller plants have a lesser amount of chlorophyll content compared to shorter and younger plants. The positive relationship between the number of pods, number of seeds, and total seed weight highlights the importance of pods and seeds to the total seed weight but not yield, as this study shows. However, hundred seed weight and seed characteristics, on the other hand, have positive impacts on yield. Hence, they are good targets for yield improvement programs. Seed characteristics contribute to the yield more than pods, basically due to postharvest factors such as damaged seeds, which is the most important factor in postharvest yield loss. This accounts for the disparity in the relationship between the number of pods, the number of seeds harvested, and the eventual yield obtained after processing of the seeds.

According to Valombola et al. (2019), similarities in accessions may result from the same accessions bearing different names due to different sources of cultivation. They, however, suggested that breeders should select accessions from different clusters to maximize heterosis. The cluster analysis shows the dendrogram which looks at the combined locations and years (Figure 3). The accessions in the first cluster (red segment) are characterized by taller plants, longer leaf length, longer leaf width, high germination rate, longer petioles, high seed weight, longer, wider, and thicker seeds, and are high yielding. The second cluster (green segment) is characterized by accessions having an early emerging date, high in chlorophyll, and flowering early, while the last cluster (blue segment) is majorly characterized by accessions having a high number of pods and number of seeds. This supports the findings of the principal component analysis.

In the correlation matrix (Figure 4), the positive correlation between the numbers of pods, number of seeds, and total seed weight indicates the importance of the number of pods and seeds on the total seed weight. However, this does not affect the yield as they are negatively correlated to the yield. The traits correlating positively with yield in this study are the same as those reported in the study of Gbaguidi et al. (2018) except for the number of seeds, which is negatively correlated in this study but positively correlated in their study. From this study, important traits for yield improvement are seed parameters and a hundred seed weight. Environmental variations also indicate the role of the environment on yield and related traits. A better understanding of the relationship between the yield-related components will provide an appropriate way of improving the yield of crops. Correlation among different components indicates the complementary functional roles of these traits on grain yield and their adaptability to different locations. The positive correlation between seed length and seed width will be crucial for farmers as they will prefer seeds with big size. Therefore, these two parameters are important for size improvement. The result from the study of Valombola et al. (2019) supports this result.

The result of the genetic parameters showed higher values for phenotypic than genotypic variances for all traits, implying the influence of the environment on the expressed traits. This result is in line with the reports of Onwubiko et al. (2019) and Khan et al. (2021). GCV and PCV values were categorized based on the suggested index of 0-10% for low, 10–20% for moderate, and ≥ 20% for high variation (Khan et al., 2020). Based on this category, genetic component analysis showed that GCV was high for number of petioles, number of pods, and hundred seed weight and yield, while PCV was high for most of the traits. Traits with reasonable variations present a wide opportunity for improvement (Onwubiko et al., 2019) while traits exhibiting low GCV and PCV show low variability, hence, they cannot be used to discriminate among the accessions. Therefore, they cannot be used for selection for crop improvement. Considering heritability and genetic advance, none of the traits in this study show both high heritability and genetic advance, which implies that direct selection cannot be recommended because of the low addictive gene effect. Low to moderate heritability and genetic advance values can hinder the use of traits for selection purposes due to high environmental effects over genetic effects (Ridzuan et al., 2019). Therefore, effective selection can be accomplished by picking traits with high GCV, PCV, heritability, and genetic advance (Usman et al., 2014). Selection of traits with low heritability and genetic progress should be delayed until their genetic effects outweigh their environmental effects (Onwubiko et al., 2019).

Furthermore, molecular characterization of the selected accessions will help to identify similarities and therefore, prevent selection of the same accessions for improvement. This will help in eliminating duplication of accessions during breeding programs. This study focuses on two locations and a few numbers of accessions. Therefore, highly contrasting environments can be used in the trial to give a more rounded and concrete locational response. Multilocational trials should be carried out on large germplasm collections to extensively detect most of the variations in BGN, followed by a selection of the best accessions as regards a trait of interest based on the objectives of the researchers to develop new and improved varieties of the crop.

## Conclusion

Expressions of genes that regulate various traits are subject to modification by the environment because different accessions show variations in their responses to different environments. In improvement programs, accessions are tested for their performance for some years and at multiple locations to select high-quality accessions. Though BGN has been reported to thrive in various weather conditions, its performance can be largely hampered by various environmental factors such as soil fertility, rainfall, temperature, and day length period. These factors constitute the effect of GEI on plant growth traits. More research on this crop will go a long way in achieving food security, especially in sub-Saharan Africa. Some accessions in this study, mostly those originating from southern Africa, show great promise for the development of varieties with improved phenotypic, vegetative, and yield traits. The performance of these accessions in both locations and their combined performances are good criteria for selection purposes. Low to moderate values for heritability and genetic advance in most traits studied indicated that selection from these accessions based on genetic effect is not yet possible.

## Data Availability Statement

The original contributions presented in the study are included in the article/Supplementary Material, further inquiries can be directed to the corresponding author/s.

## Author Contributions

OSO, OO, and MA designed the experiment. OSO carried out the trials, collected the data, performed the analysis, and wrote the manuscript. OOB, OO, and MA supervised the study and reviewed the manuscript. All authors contributed to the article and approved the submitted version.

## Conflict of Interest

The authors declare that the research was conducted in the absence of any commercial or financial relationships that could be construed as a potential conflict of interest.

## Publisher's Note

All claims expressed in this article are solely those of the authors and do not necessarily represent those of their affiliated organizations, or those of the publisher, the editors and the reviewers. Any product that may be evaluated in this article, or claim that may be made by its manufacturer, is not guaranteed or endorsed by the publisher.
