# Supplementary material for: Genetic Diversity and Environmental Influence on Growth and Yield Parameters of Bambara Groundnut
Source: Front Plant Sci. 2021 Dec 20;12:796352. doi: 10.3389/fpls.2021.796352 (PMC8721115; doi:10.3389/fpls.2021.796352)
Supplement: Supplementary file 3 [file Presentation_1.PPTX]

## Slide 1
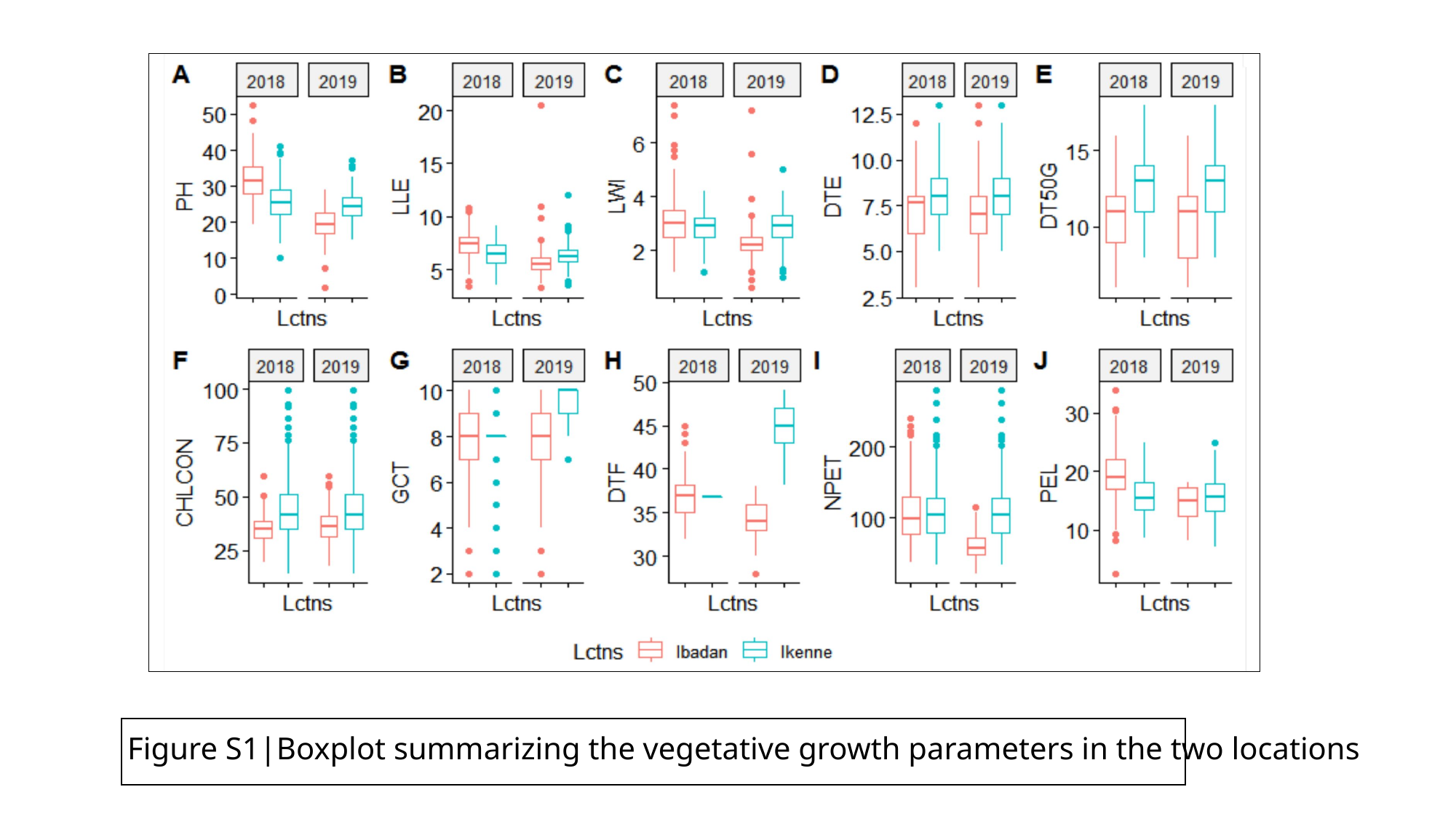

# Figure S1|Boxplot summarizing the vegetative growth parameters in the two locations

## Slide 2
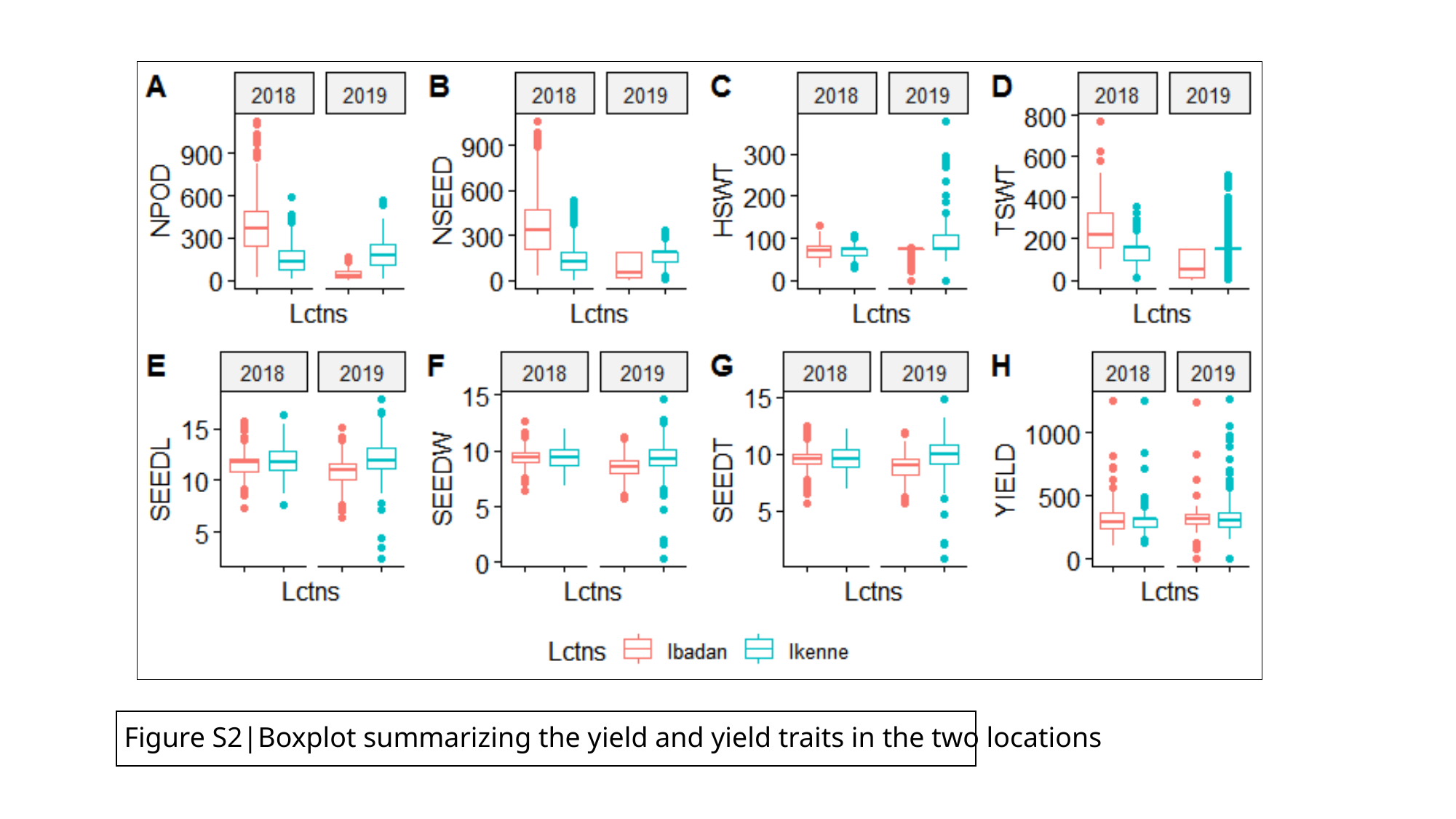

# Figure S2|Boxplot summarizing the yield and yield traits in the two locations

## Slide 3
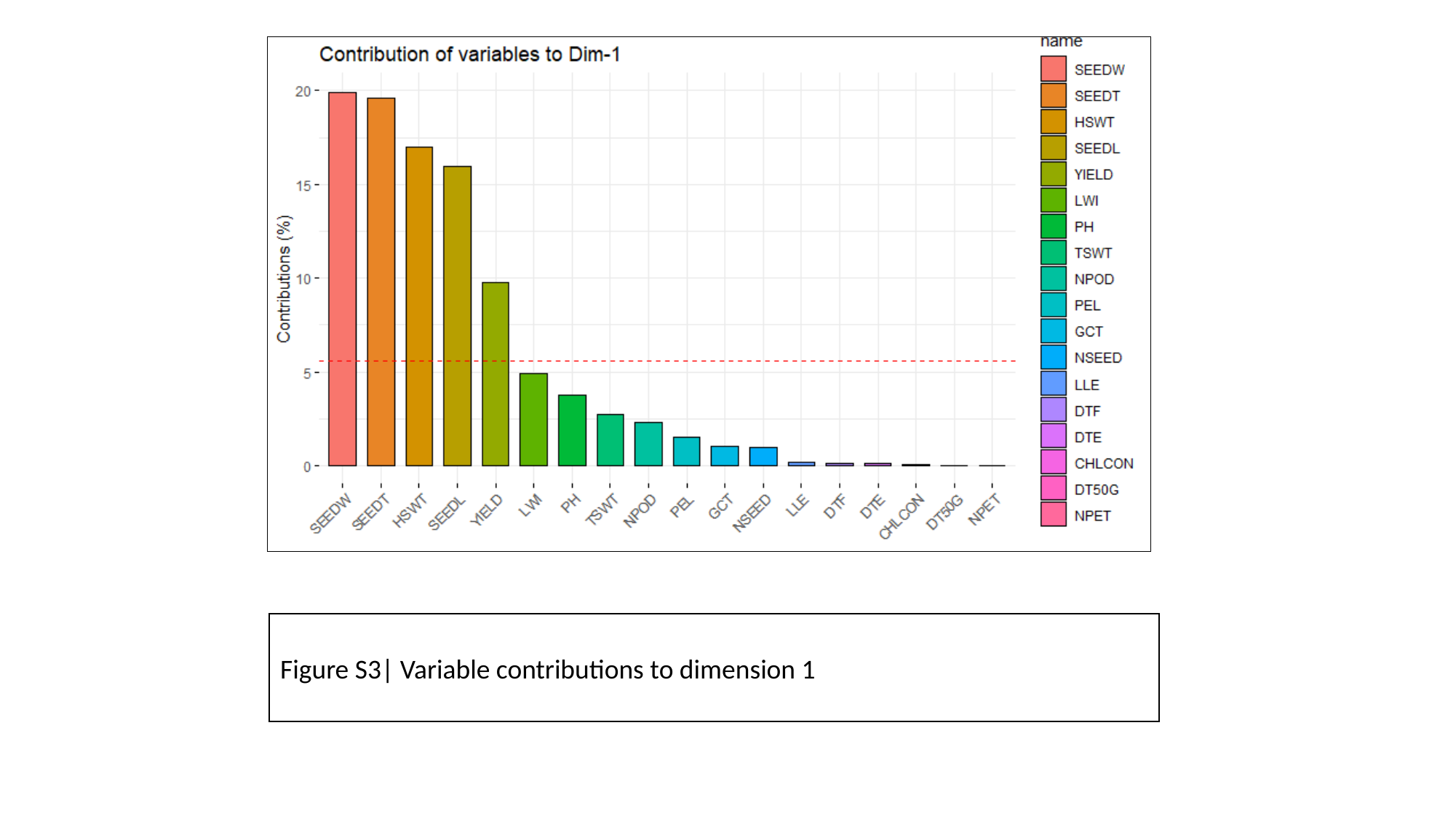

Figure S3| Variable contributions to dimension 1

## Slide 4
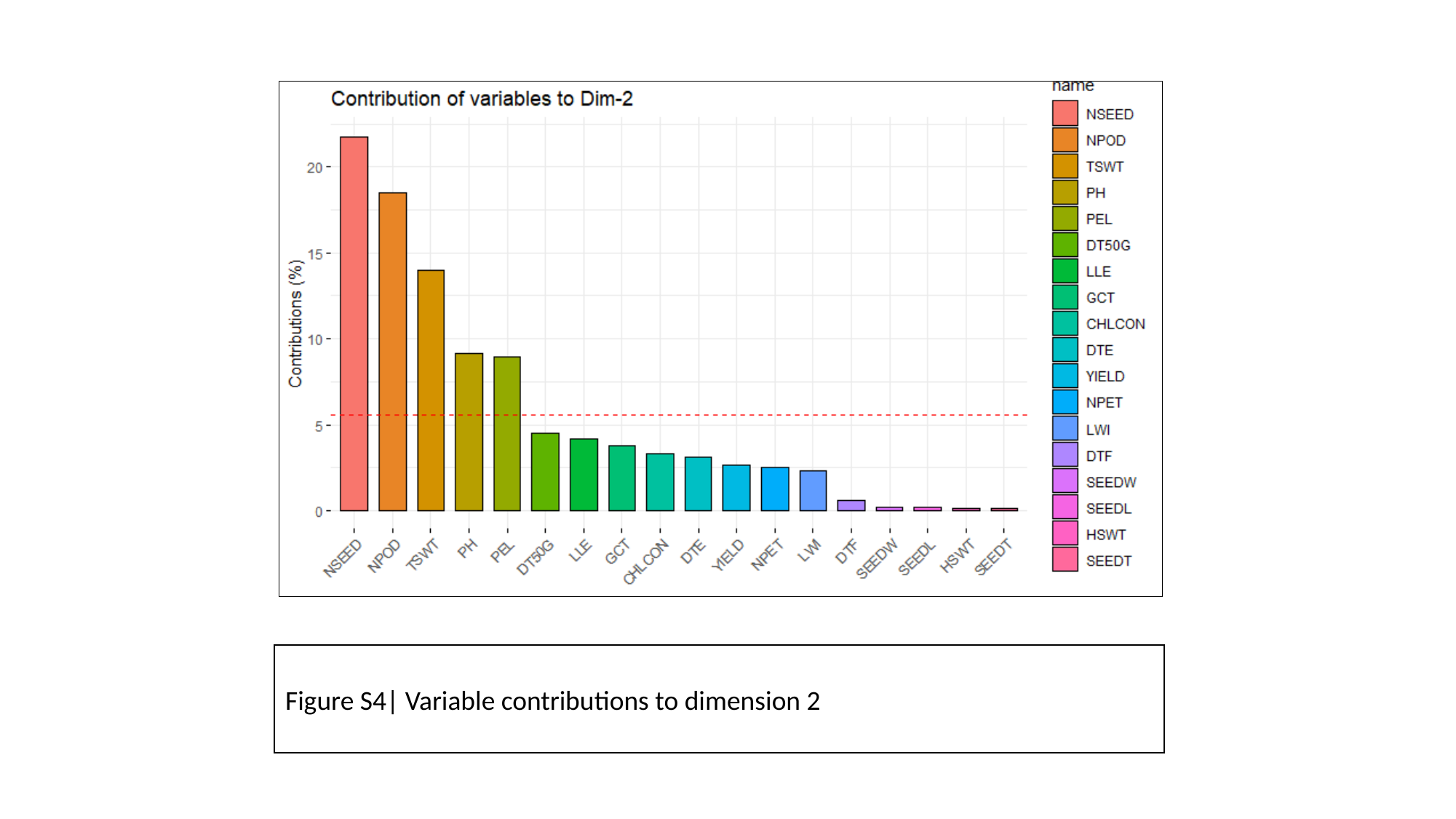

Figure S4| Variable contributions to dimension 2

## Slide 5
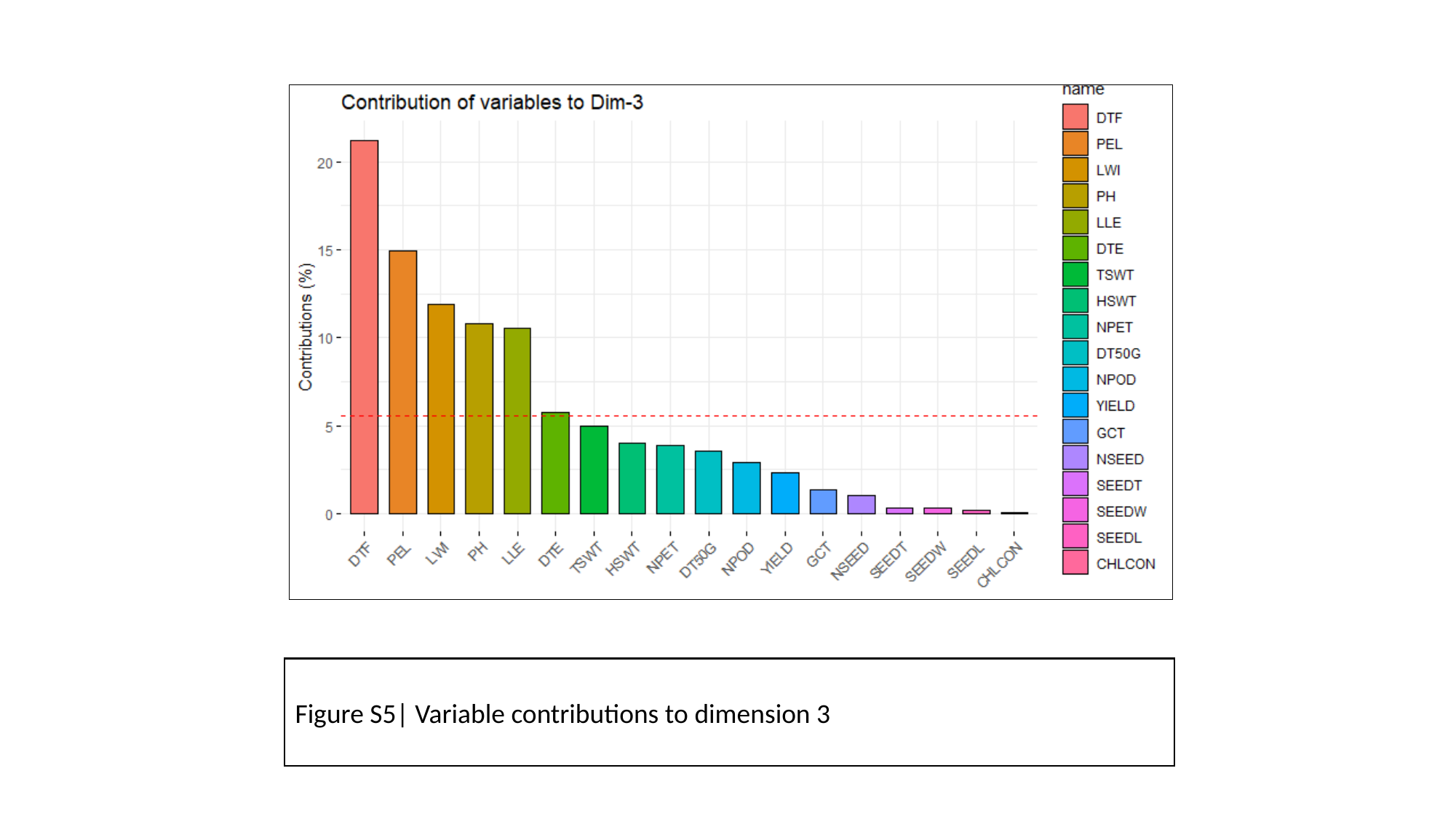

Figure S5| Variable contributions to dimension 3

## Slide 6
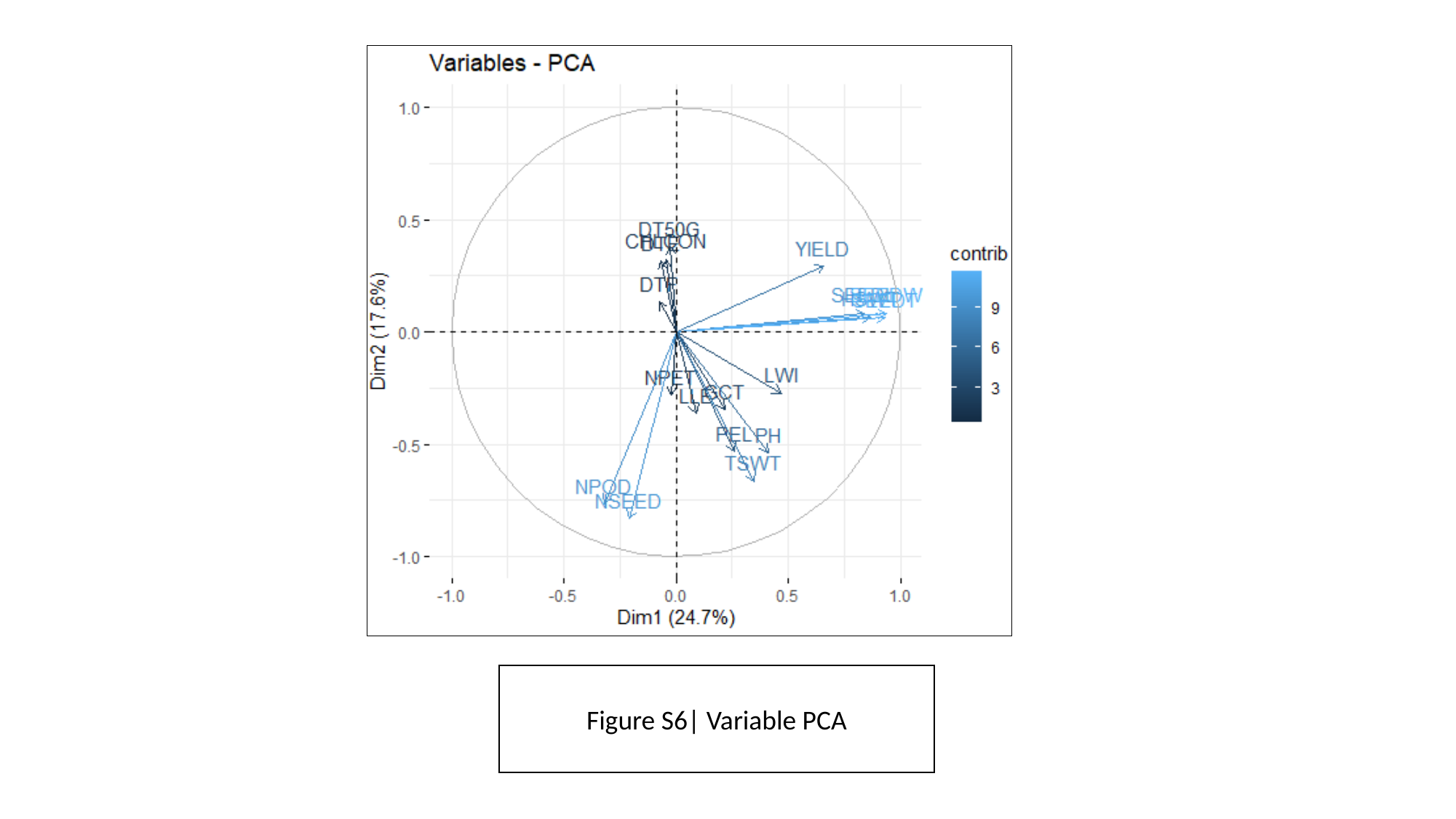

Figure S6| Variable PCA

## Slide 7
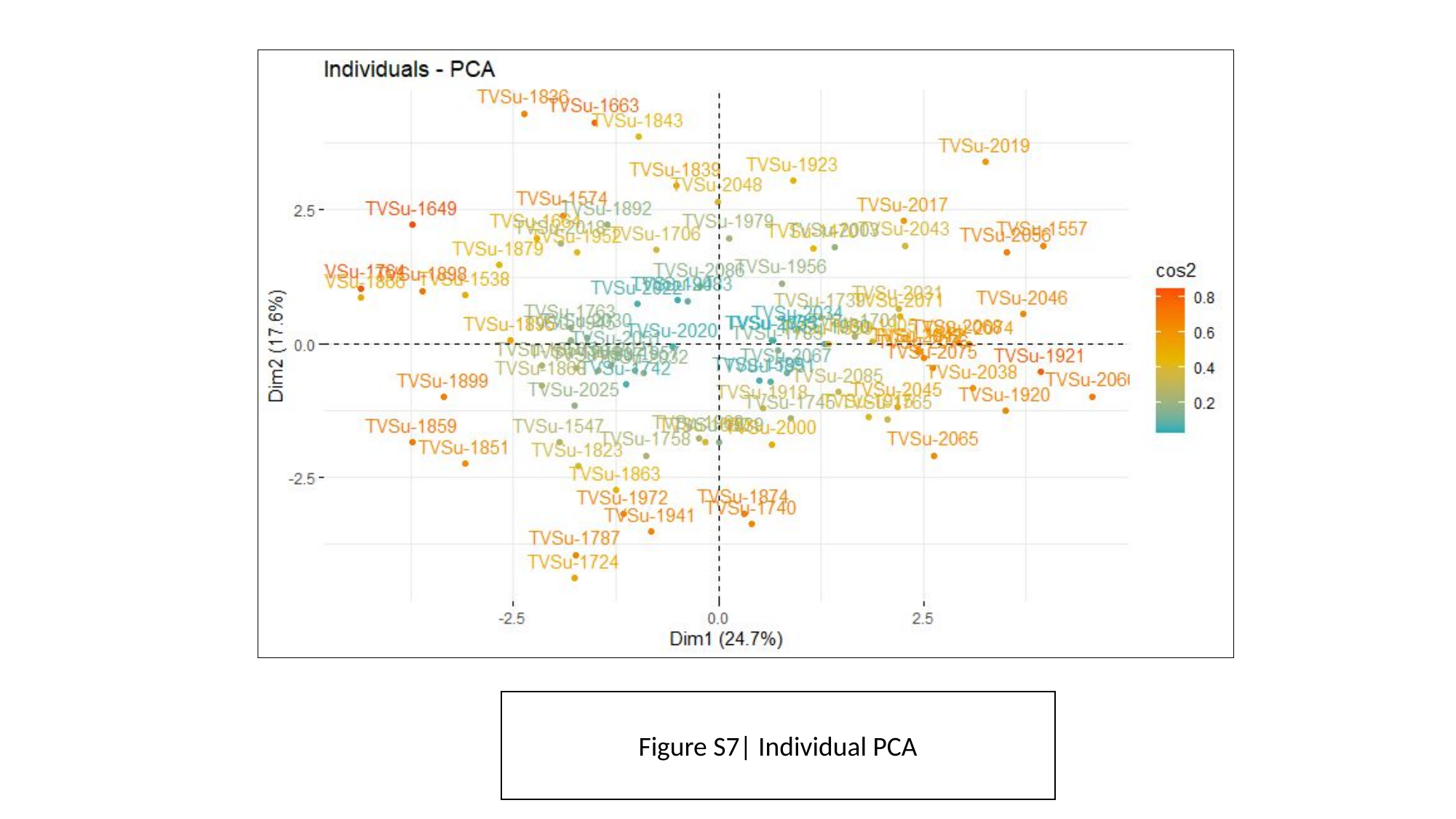

Figure S7| Individual PCA
